# Anatomically‐designed bicruciate‐retaining total knee arthroplasty demonstrates good two‐year survivorship and improvements in patient‐reported outcomes: A prospective multicenter single‐arm study

**DOI:** 10.1002/jeo2.70567

**Published:** 2025-11-14

**Authors:** Ryota Yamagami, Osamu Nishiike, Ayano Kuwasawa, Katsuhisa Ishii, Daisuke Hamada, Keinosuke Ryu, Shigeshi Mori, Kotaro Yamagishi, Sarah Megginson, Masao Akagi

**Affiliations:** ^1^ Department of Orthopaedic Surgery Faculty of Medicine The University of Tokyo Tokyo Japan; ^2^ Department of Orthopedics Kushiro Sanjikai Hospital Kushiro Hokkaido Japan; ^3^ Department of Orthopedics Saitama Cooperative Hospital Kawaguchi Saitama Japan; ^4^ Department of Orthopedics Toho University Ohashi Medical Center Tokyo Japan; ^5^ Department of Orthopedics, Institute of Biomedical Sciences Tokushima University Graduate School Tokushima Japan; ^6^ Department of Orthopedics Nihon University Itabashi Hospital Tokyo Japan; ^7^ Department of Orthopedic Surgery Kindai University Nara Hospital Ikoma Nara Japan; ^8^ Department of Orthopedics Kindai University Hospital Osaka Japan; ^9^ Global Data Analytics, Global Clinical Research Operations Smith & Nephew Hull UK; ^10^ Department of Orthopedic Surgery Center of Joint Replacement Surgery, Kashimoto Hospital Osaka Japan

**Keywords:** bicruciate‐retaining, implant survival rate, patient‐reported outcome measures, range of motion, Total knee arthroplasty

## Abstract

**Purpose:**

To evaluate the early safety and performance of a novel anatomical‐design bicruciate‐retaining total knee arthroplasty (BCR‐TKA) implant (JOURNEY™ II XR; JIIXR) in a prospective multicenter single‐arm cohort, with 2‐year implant survivorship as the prespecified primary endpoint, and patient‐reported outcomes and radiographic findings as secondary exploratory endpoints.

**Methods:**

Ninety‐four patients (106 knees) underwent cemented BCR‐TKA with JIIXR at eight centers. The primary endpoint was 2‐year survivorship tested for noninferiority versus a 98% benchmark using exact binomial (one‐sided 97.5% CI) and Kaplan–Meier methods. Secondary endpoints included the EuroQol 5‐Dimension 5‐Level (EQ‐5D‐5L) questionnaire, its visual analogue scale (EQ‐VAS), Forgotten Joint Score‐12 (FJS‐12, collected postoperatively only), Knee Injury and Osteoarthritis Outcome Score (KOOS), range of motion, and radiolucent lines assessed in standardized zones.

**Results:**

At 2 years, survivorship was 97.9% (94/96), confirming non‐inferiority (*p* = 0.0064). Patient‐reported outcome measures improved over follow‐up: EQ‐VAS increased from 62.0 preoperatively to 83.7 at 2 years; EQ‐5D‐5L index from 0.61 to 0.84; and KOOS pain and symptoms from 52.8 and 59.2 to 87.9 and 84.6, respectively. FJS‐12 increased from 40.0 at 3 months to 65.4 at 2 years. Across measures, the changes exceeded published minimally clinically important differences (MCIDs): all KOOS subscales met MCID by 1 year, EQ‐VAS exceeded MCID by 3 months, and FJS‐12 exceeded MCID between 3 months and 1 year. Radiolucent lines were observed in 35 knees (36.5%); total length decreased from 5.1 ± 6.7 mm at 3 months to 2.6 ± 2.3 mm at 2 years with no radiographic loosening. Range of motion at 2 years was similar to baseline.

**Conclusions:**

In this prospective single‐arm multicenter study, an anatomical‐design BCR‐TKA implant achieved good 2‐year survivorship and clinically meaningful improvements in patient‐reported outcomes, with stable radiographic findings. These data support early safety and performance while underscoring the need for long‐term comparative studies to establish durability and relative effectiveness.

**Level of Evidence:**

Level II, therapeutic study.

**Abbreviations**
ADL: activities of daily living
AP: anteroposterior
BCR: bicruciate‐retaining
BMI: body mass index
CI: confidence interval
EQ‐5D‐5L: EuroQol Five Dimensions Questionnaire
EQ‐VAS: EuroQol Visual Analogue Scale
FJS‐12: Forgotten Joint Score‐12
JIIXR: JOURNEY™ II XR
KOOS: Knee Injury and Osteoarthritis Outcome Score
MCIDs: minimally clinically important differences
PROMs: patient‐reported outcome measures
QOL: quality of life
RLLs: radiolucent lines
ROM: range of motion
TKA: total knee arthroplasty
VAS: visual analogue scale
XLPE: crosslinked polyethylene

## INTRODUCTION

Total knee arthroplasty (TKA) is widely recognized as the standard treatment for end‐stage osteoarthritis as it effectively alleviates pain and restores joint function [30, 36]. Worldwide, the number of TKAs has increased steadily, reflecting socioeconomic factors and advances in surgical and implant technology [22]. Long‐term outcomes are generally reliable, with substantial improvements in mobility and quality of life [14]; nevertheless, only 70%–80% of patients report satisfaction, indicating persistent unmet functional expectations [7, 18]. As such, further refinements in implant design and surgical techniques are warranted.

Bicruciate‐retaining TKA (BCR‐TKA) is designed to reproduce native knee kinematics by preserving both cruciate ligaments. Early iterations were limited by technical challenges and inconsistent outcomes [1, 6], however, advancements in implant design and surgical tools have reignited interest in this area. Recent studies with BCR‐TKA have demonstrated more physiological knee kinematics [19, 38], enhanced proprioception [3], and improved joint stability [21] versus cruciate‐sacrificing designs (including both posterior cruciate‐retaining or substituting TKA [23, 33]). These studies have shown anatomical implant geometry is central to reproducing near‐native kinematics [35].

The JOURNEY^TM^ II XR (JIIXR) Total Knee System (Smith & Nephew) is a newly developed anatomical BCR‐TKA implant incorporating asymmetric condylar geometry and a three‐degree medial joint‐line inclination. In vivo studies have demonstrated near‐physiological kinematics with this design [20], yet prospective clinical evidence on early safety and performance remains limited.

Therefore, this study was designed as a prospective multicenter postmarket investigation to evaluate the early safety and performance of the JIIXR. Given the historical early mechanical complications with some BCR designs, an a priori focus on early survivorship was adopted. This single‐arm investigation evaluated 2‐year implant survivorship tested for noninferiority against a predefined performance goal of 98% [32], with patient‐reported outcomes (PROMs) and radiographic findings as secondary exploratory endpoints. It was hypothesized that 2‐year survivorship would be noninferior to the 98% benchmark (margin, 7%), that PROMs would improve over the follow‐up period, and that radiographic assessments would not indicate any early loosening.

## METHODS

### Study design and ethics approval

This prospective, multicenter, postmarket clinical follow‐up, single‐arm study was conducted at eight centers across our country. Up to eight clinical sites were required to enrol the target sample size over approximately 24 months. All participating surgeons were experienced in implanting the JIIXR Total Knee System, with documented training and expertise in the surgical procedure before participation. The protocol was approved by the ethics committees of all participating institutions, and all participants provided written informed consent prior to enrolment. The study protocol complied with the Ethical Guidelines for Medical and Health Research involving Human Subjects and the principles of the Declaration of Helsinki.

### Participants

Patients who met all inclusion and exclusion criteria, as determined by the participating surgeons, were prospectively enroled between 15 November 2018 and 19 February 2021. Eligibility criteria included: a diagnosis of arthritis (rheumatoid, posttraumatic or degenerative) or failed previous surgeries necessitating TKA, intact cruciate and collateral ligaments in the affected joint, age ≥20 years, and the determination by participating surgeons that the JIIXR Knee was the optimal treatment device. Patients were excluded if they met any of the following criteria: corticosteroid intra‐articular therapy within the past 3 months, severe deformities (varus/valgus >15°), prior TKA, unresolved or poorly functioning prior TKA or unicompartmental knee arthroplasty in the contralateral knee, unresolved prior hip arthroplasty or severe hip arthritis, conditions known to negatively affect TKA outcomes (such as Paget's disease, Charcot's disease, uncontrolled diabetes, immunosuppressive disorders or active infections), and body mass index (BMI) > 40 kg/m².

### Study device and surgical procedure

**Figure 1:**
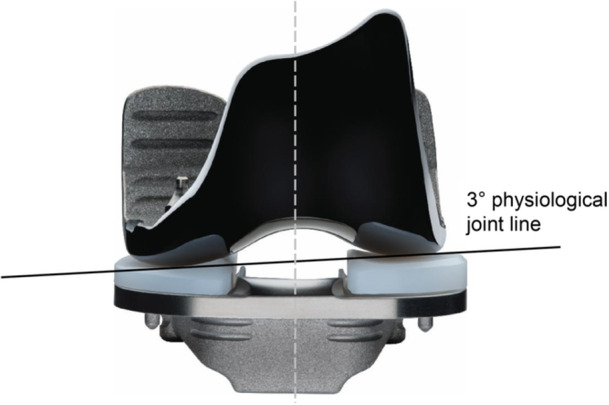
JOURNEY II XR Total Knee System.

All patients underwent cemented BCR‐TKA using the JIIXR Total Knee System. Unlike conventional TKA devices, the JIIXR implant features a three‐degree medial inclination in the joint line. This reflects the asymmetrical geometry of the medial and lateral condyles to mimic the knee's native anatomy (Figure 1). Its horseshoe‐shaped tibial baseplate allows the attachment site of both cruciate ligaments to be preserved. The femoral component is manufactured from oxidized zirconium (OXINIUM^TM^; Smith & Nephew), and the tibial insert from crosslinked polyethylene (XLPE). The patellar component is made of ultra‐high molecular weight polyethylene or XLPE, and the tibial base of a titanium 6Al‐4V alloy. All study‐related procedures were conducted following the manufacturer's recommended technique, including standard medial arthrotomy and patella resurfacing. Bone resection was performed using mechanical or functional alignment strategies, with either manual instruments or an image‐free navigation system. Alignment strategy was chosen at the surgeon's discretion. Functional alignment was used in 28 of 106 enroled knees (26%), with mechanical alignment used in the remaining knees. The chosen alignment strategy was recorded intraoperatively. Postoperative rehabilitation was not standardized across sites. In general, early mobilization was implemented with physical therapy focusing on knee range‐of‐motion, quadriceps activation/strengthening, gait training, and progressive weight‐bearing as tolerated, with home exercises and outpatient therapy.

### Endpoints

The primary endpoint was the implant survival rate of the JIIXR Total Knee System at 2 years postimplantation. Implant survivorship was defined as the absence of revision or removal of any device component.

The secondary endpoints included PROMs, including the EuroQol Five Dimensions Questionnaire (EQ‐5D‐5L), Forgotten Joint Score (FJS‐12), and Knee Injury and Osteoarthritis Outcome Score (KOOS). Radiographic outcomes (mechanical alignment, radiolucencies) and knee range of motion (ROM) were also collected. EQ‐5D‐5L, KOOS and ROM were assessed preoperatively and at 3 months, 6 months, 1 year and 2 years postoperatively. FJS‐12 was evaluated at 3 months, 6 months, 1 year and 2 years postoperatively.

#### EuroQol Five Dimensions Questionnaire

The EQ‐Visual Analog Scale (VAS) captured the respondents' self‐assessed health on a vertical scale ranging from ‘Best imaginable health state’ to ‘Worst imaginable health state’. When combined with the EQ‐5D‐5L score (activity, anxiety, mobility, pain and self‐care), the data provide quantitative health outcome measures [13]. The EQ‐VAS score is rated on a scale of 0–100, with 100 representing the best health and 0 the worst. The EQ‐5D‐5L combined score ranges from 0 to 1, with higher scores indicating better health.

#### Forgotten Joint Score

The FJS‐12 is a PROM that assesses joint awareness during the activities of daily living. Joint awareness can be defined as any unintended perception of a joint [4]. It comprises 12 questions scored on a 0–100 scale, with higher scores reflecting reduced joint awareness and greater ability to forget the affected/replaced knee during daily living activities.

#### Knee Injury and Osteoarthritis Outcome Scores

The KOOS assesses the individual's perception of their knee and knee‐related issues. It evaluates the short‐ and long‐term effects of knee injury and the implications of primary osteoarthritis. It includes five subscales: pain, other symptoms, activities of daily living, sport and recreation function and knee‐related quality of life [27]. Each subscale is scored from 0 to 100, with 0 representing extreme problems and 100 representing no problem.

#### ROM

ROM was measured by the single principal investigator in each participating institution using a goniometer, which evaluated flexion, extension, and total ROM (flexion plus extension). Measurements were taken preoperatively and at 3 months, 6 months, 1 year, and 2 years postoperatively.

#### Radiographical Assessment

**Figure 2:**
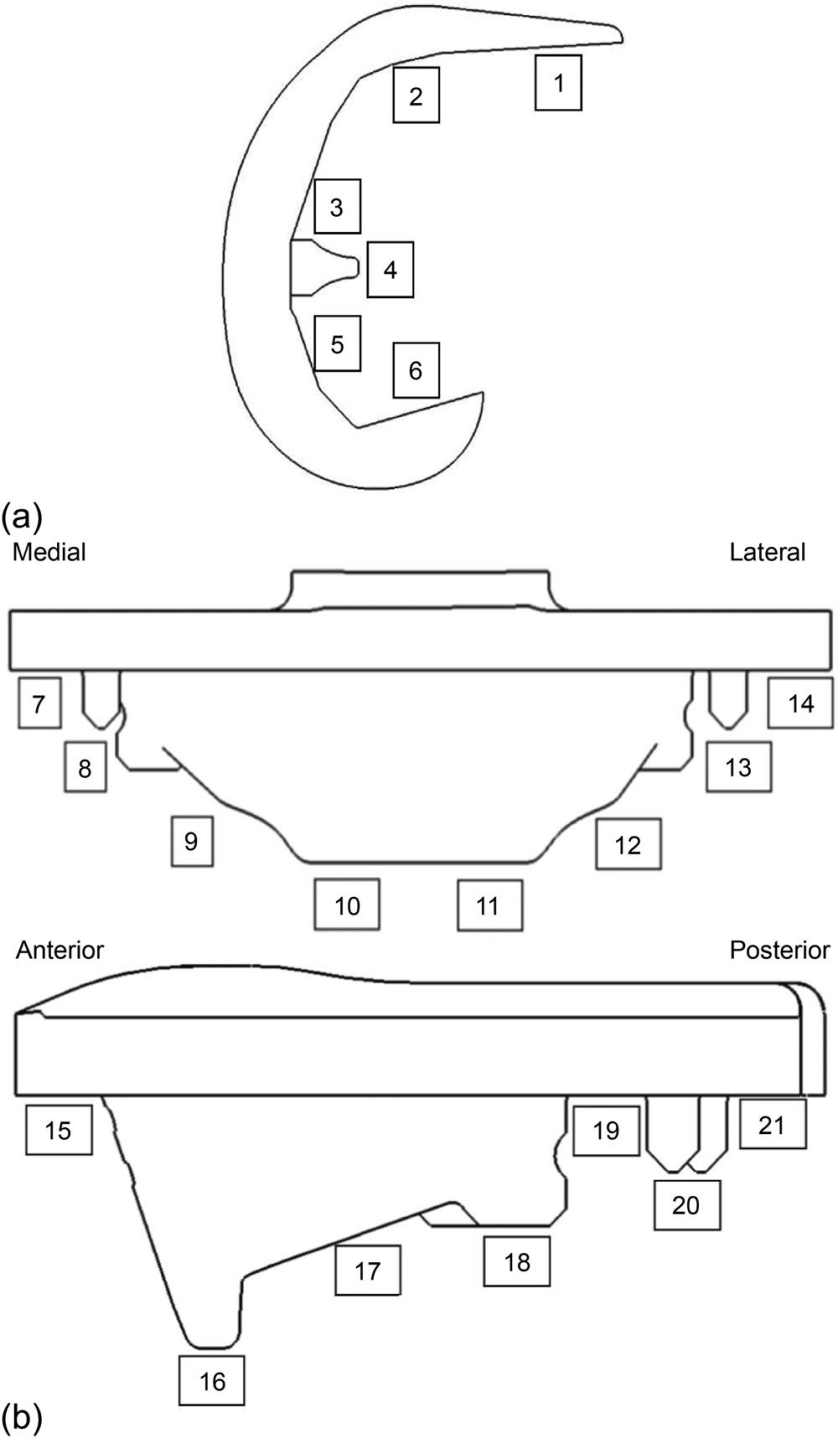
Zone of radiolucent lines. (a) Lateral view of femoral implant. (b) Anteroposterior and lateral view of tibial implant.

The following radiographs were obtained: (1) Standard anteroposterior (AP) long‐leg view (at the preoperative visit within 6 months before the surgery and at discharge) for measuring lower limb alignment; (2) standard AP view (weight‐bearing or supine) and lateral view (at operation/discharge and at 3 months, 6 months, 1 year, and 2 years postoperatively) for evaluating the presence of radiolucent lines (RLLs) in each zone (Figure 2) and if present, measuring their total length [2].

### Statistical analysis

The sample size was powered to evaluate noninferiority of the 2‐year survival rate against a performance goal of 98%, with a noninferiority margin of 7%. The 98% performance goal was based on a contemporary registry‐derived 2‐year survivorship rate of modern TKA [32]. The noninferiority margin was set at 7% to preserve a clinically meaningful fraction of this benchmark while maintaining feasibility for a single‐arm observational evaluation. Noninferiority required the lower bound of the one‐sided 97.5% confidence interval (CI) for the 2‐year survivorship to exceed 91% (98%−7%). This approach follows regulatory guidance for noninferiority assessments that recommend prespecifying margins using external evidence and clinical judgement to preserve a fraction of the control effect (e.g., International Council for Harmonisation of Technical Requirements for Pharmaceuticals for Human Use E10; U.S. Food and Drug Administration Guidance for Industry on Non‐Inferiority Clinical Trials). A sample size of 100 knees was required to achieve 80% power for testing the primary hypothesis using a one‐sided 97.5% CI calculated via exact binomial methods. Considering an expected drop‐out rate of approximately 5% over 2 years, 94 participants (106 knees) were enroled.

Data were collected preoperatively, intraoperatively, at discharge, and at 3 months, 6 months, 1 year and 2 years postoperatively. A final analysis was performed after all enroled participants completed their 2‐year postoperative visits. Statistical analyses were conducted by Smith & Nephew's Global Biostatistics group using SAS Software, version 9.4 (SAS Institute). Unless otherwise stated, all significance and hypothesis testing were two‐sided and performed at the 5% significance level. Where applicable, resulting *p*‐values and 95% two‐sided CIs were reported. Categorical variables are summarized as frequencies and percentages. Continuous variables are summarized with mean and standard deviation. For the primary outcome, the device survivorship proportion at two years was the endpoint of interest, analyzed using Binomial Clopper‐Pearson method and Kaplan–Meier estimates. For the purpose of estimating implant survivorship, subjects who discontinued prior to completion of the study, or were lost to follow‐up, had their data censored on the date of discontinuation/last known study visit date. The secondary outcomes are summarized with appropriate descriptive statistics for continuous or categorical variables, no imputation for missing data was made. The incidence and number of participants reporting device deficiencies are also summarized. For the analysis of secondary endpoints, preoperative and postoperative measures were compared using paired t‐tests. Secondary endpoints (PROMs, ROM and radiographic measures) were prespecified as exploratory. Given the multiplicity of outcomes and time points, no formal adjustment for multiple comparisons was applied; *p*‐values for secondary analyses are nominal and interpreted descriptively. Radiographic outcomes were summarized descriptively (including zone‐level frequencies and total RLL length per knee); no hypothesis testing was performed at the zone level.

## RESULTS

**Figure 3:**
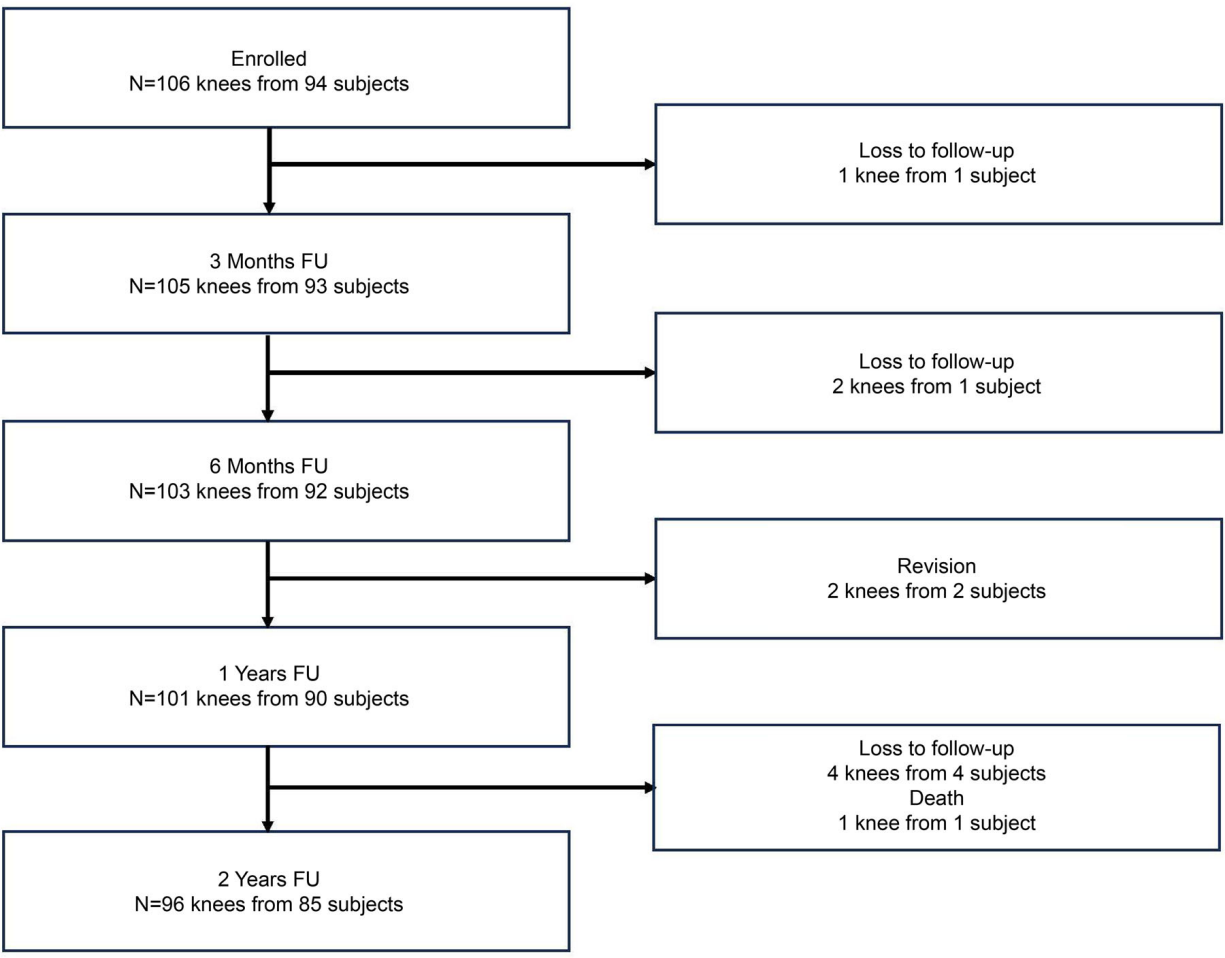
Flowchart demonstrating the disposition of the participants, including the total distribution of enroled, evaluated and safety‐evaluated participants. FU, follow‐up.

A total of 94 participants (106 knees) were enroled between November 2018 and February 2021. Nine participants (10 knees) were discontinued from the study for the following reasons: death (one participant/one knee), revision (two participants/two knees) and loss to follow‐up (six participants/seven knees). The flow of participant enrolment and analysis is shown in Figure 3, and baseline demographic characteristics are summarized in Table 1.

**Table 1:** Baseline characteristics of participants.

Demographic	Subjects (*n* = 94)
Age (years)	
Unilateral/bilateral	71.7 ± 6.8
Unilateral	82 (87.2%)
Bilateral	12 (12.8%)
Knee side	
Left knee	48 (45.3%)
Right knee	58 (54.7%)
Age group	
<65 years	3 (3.2%)
≥65 years	91 (96.8%)
Gender	
Female	68 (72.3%)
Male	26 (27.7%)
Race/ethnicity	
Japanese	93 (98.9%)
Other Asian	1 (1.1%)
Primary diagnosis	
Osteoarthritis	105 (99.1%)
Rheumatoid arthritis	1 (0.9%)

*Note*: Data are presented as mean ± standard deviation or number (percentage).

### Primary endpoint

Two revisions occurred during the study period, revealing a 2‐year survival rate of 97.9% (94/96) (95% CI 92.7%–99.8%). One participant's implant (right knee) was revised at 13.1 months due to knee instability and lateral dislocation. Prerevision long‐leg and component radiographs demonstrated neutral mechanical alignment and appropriate component positioning without radiographic loosening. In this case, the tibial baseplate and liner were removed and revised to a cruciate‐retaining implant. Another participant's implant (left knee) was revised to a cruciate‐retaining implant at 11.1 months for progressive, unexplained pain refractory to non‐operative management. Pre‐revision clinical and radiographic assessment showed no loosening or instability. Pain improved after revision. Both participants were women aged 82 and 72 years, respectively.

**Figure 4:**
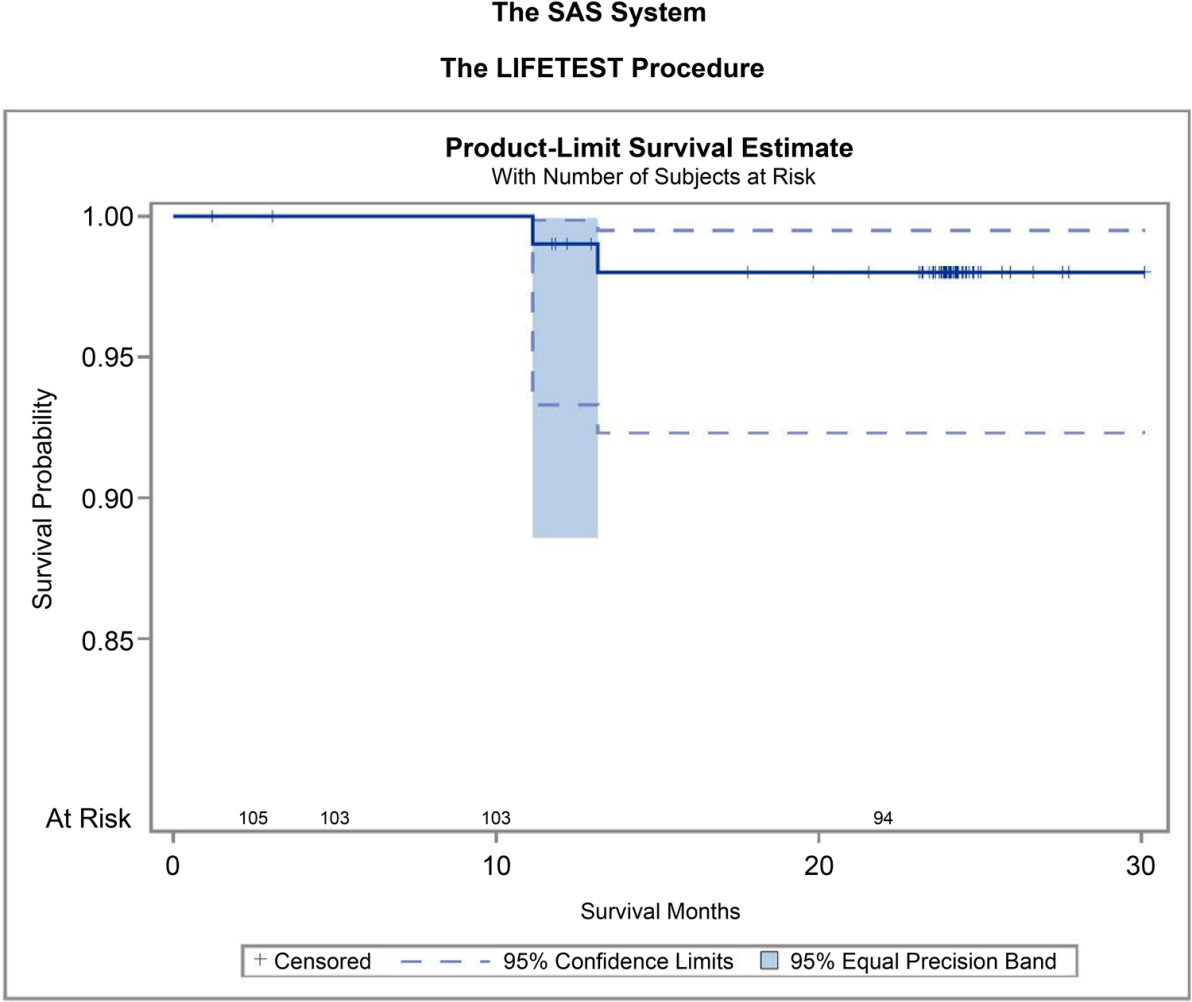
Kaplan–Meier implant survival curve until 2 years postoperatively.

Noninferiority relative to the performance goal was assessed using the Clopper–Pearson and Kaplan–Meier methods. This analysis confirmed that the JIIXR Total Knee System was no worse than the 98% performance goal with a margin of 7% (91% noninferiority limit, *p* = 0.006). Of the 96 knees analyzed, 94 (97.9%) remained intact after 22 months of implantation (95% CI, 92.68–99.75), with a noninferiority limit of 91% (*p* = 0.0064). Figure 4 shows the Kaplan–Meier implant survival curve up to 2 years postoperatively.

### Secondary endpoints

#### Patient‐reported outcome measures

**Figure 5:**
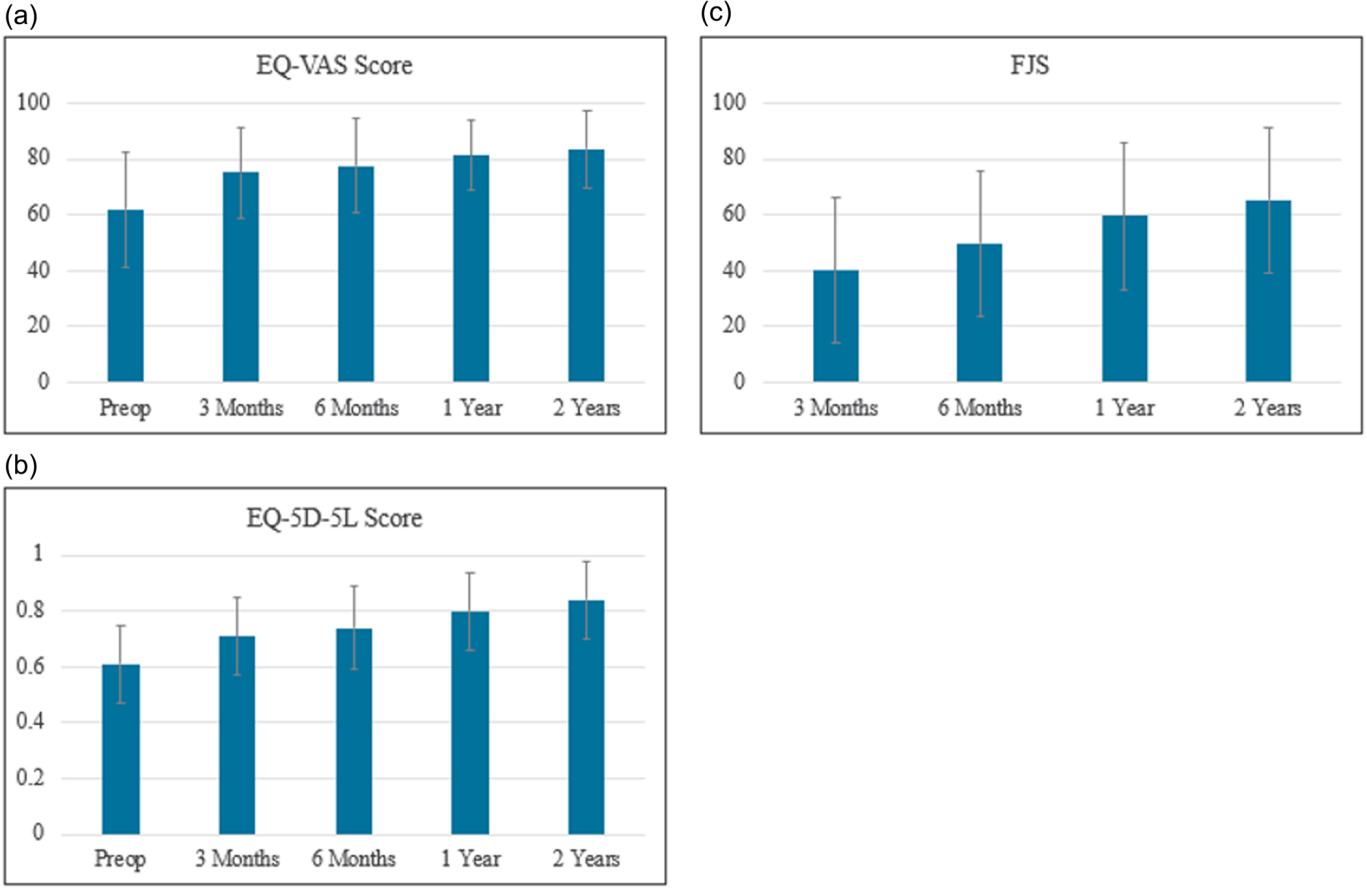
Patient‐reported outcome measures before and after JOURNEY II XR. (a) EQ‐VAS Score. Significant improvements in EQ‐VAS score were observed at all postoperative assessments. (b) EQ‐5D‐5L Score. Significant improvements in EQ‐5D‐5L score were observed at all postoperative assessments. (c) Forgotten Joint Score. The score at the three‐month visit was 40.0 ± 26.0, and it had increased to 65.4 ± 25.9 by the 2‐year assessment. EQ‐VAS, EuroQol Visual Analogue Scale; EQ‐5D‐5L, EuroQol Five Dimensions Questionnaire; FJS, Forgotten Joint Score.

**Figure 6:**
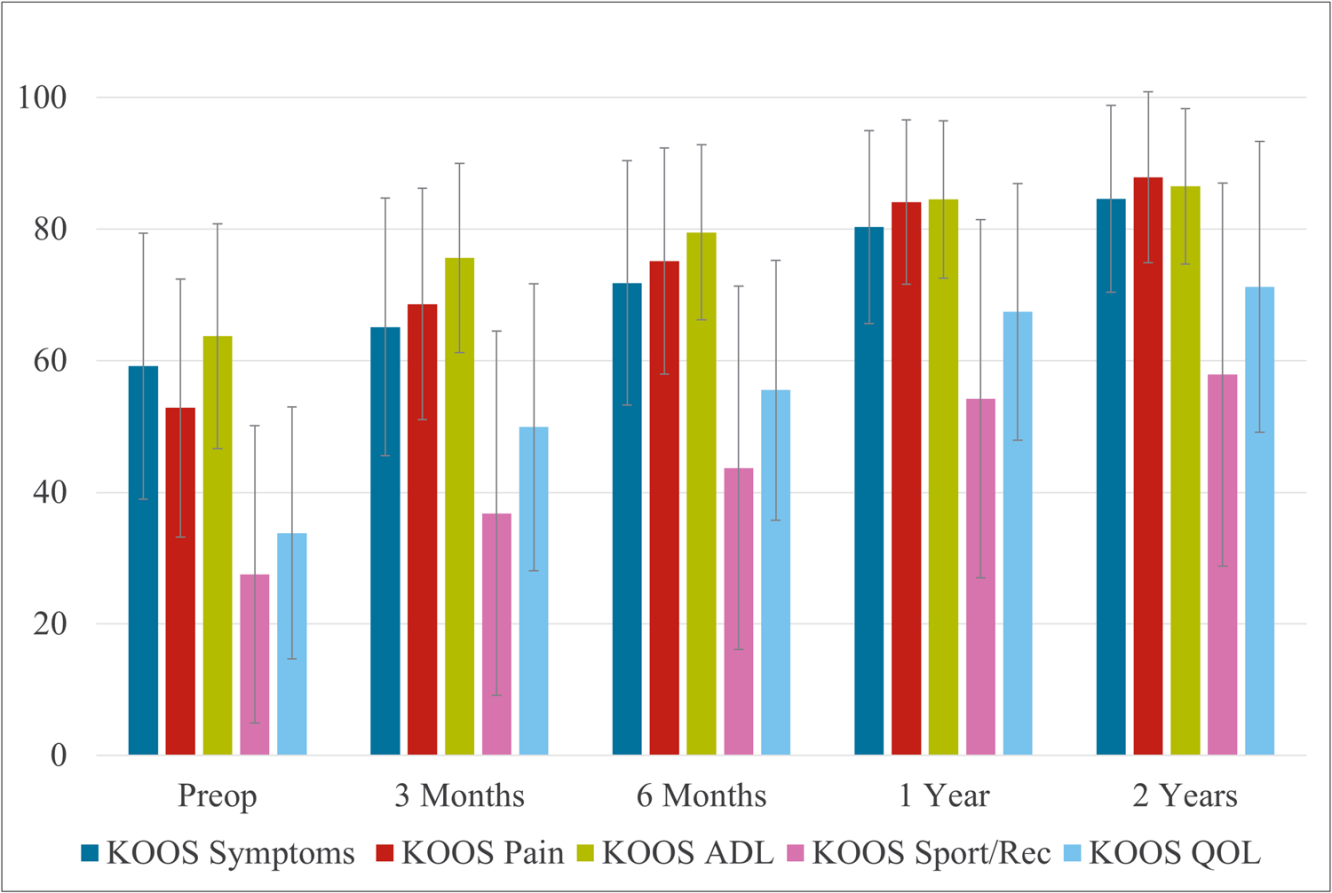
Knee Injury and Osteoarthritis Outcome Score before and after JOURNEY II XR. Significant increases in scores were observed for all five KOOS subscales. ADL, Activities of Daily Living; KOOS, Knee Injury and Osteoarthritis Outcome Scores; QOL, Quality of Life.

The results for the PROMs are presented in Figures 5 and 6. At the preoperative visit, the EQ‐VAS score (mean ± standard deviation) was 62.0 ± 20.7, which improved significantly to 83.7 ± 13.8 by the 2‐year assessment (Figure 5a). The mean EQ Health score at the preoperative visit was 0.61 ± 0.14, which increased to 0.84 ± 0.14 by the 2‐year assessment (Figure 5b). Significant improvements in EQ‐VAS and EQ Health scores were observed at all postoperative assessments (*p* < 0.001 for both measures).

At the preoperative visit, the KOOS values for symptoms and pain were 59.2 ± 20.2 and 52.8 ± 19.6, which increased to 84.6 ± 14.2 and 87.9 ± 13.0, respectively, by the 2‐year assessment. Moreover, at all postoperative assessments, significant increases in scores were observed for all five KOOS subscales (*p* < 0.05 for all assessment points) (Figure 6).

FJS‐12 scores were collected postoperatively only. The score at the 3‐month visit was 40.0 ± 26.0, and it had increased to 65.4 ± 25.9 by the 2‐year assessment (Figure 5c).

To facilitate clinical interpretation, we compared these changes in PROMs with published minimally clinically important differences (MCIDs). All five KOOS subscales met or exceeded their respective MCIDs by the 1‐year visit (Pain ≥ 13.4, Symptom ≥ 15.4, ADL ≥ 15.4, Sports/Recreation ≥ 19.6 and QOL ≥ 21.1) [11]; the EQ‐VAS index surpassed an MCID of 6.41 [39] as early as 3 months and remained above this threshold thereafter; and the FJS‐12 improved by 19.6 points from 3 months to 1 year—exceeding the reported MCID range of 10.8–13.7—and continued to increase to 65.4 at 2 years [9, 15].

#### ROM

The ROM values for flexion, extension, and the total ROM (flexion + extension) before and after JIIXR are summarized in Table 2. Flexion had a value of 135.0 ± 10.3° preoperatively and 130.3 ± 11.4° at 2 years. Extension had a mean value of –4.0 ± 6.0° preoperatively and –1.5 ± 3.0° at 2 years. The total ROM had a value of 130.9 ± 12.3° preoperatively and 128.8 ± 11.9° at 2 years. For total ROM, statistically significant decreases from preoperative levels were seen at 3 months (*p *< 0.001), 6 months (*p* < 0.001) and 1 year (*p* = 0.013), but at 2 years, the difference was not statistically significant (*p* = 0.327).

**Table 2:** Knee range of motion before and after JOURNEY II XR.

Follow‐up	Preop	3 Months	6 Months	1 Year	2 Years
ROM Flexion	135.0 ± 10.3	125.1 ± 13.8	126.8 ± 13.6	128.8 ± 11.7	130.3 ± 11.4
ROM Extension	−4.0 ± 6.0	−2.4 ± 4.8	−2.0 ± 4.4	−1.8 ± 3.0	−1.5 ± 3.0
Total ROM	130.9 ± 12.3	122.6 ± 14.7	124.8 ± 14.0	127.0 ± 12.2	128.8 ± 11.9

Abbreviation: ROM, Range of Motion.

#### Radiographic outcomes

The mechanical alignment angle (relative to 180°) at preoperative screening was 6.4 ± 4.6° varus. At discharge, it was 1.4 ± 3.0° varus, and the range of mechanical alignment was within ±3° in 82 (77.4%) knees.

At the 3‐month assessment, 28 knees had RLLs within at least one zone of zones 1–21, and the length of the RLLs (mean ± standard deviation per knee) was 5.1 ± 6.7 mm. At the 2‐year assessment, 35 knees had RLLs within at least one zone, and the mean length of the RLLs (per knee) had decreased to 2.6 ± 2.3 mm.

At the 24‐month assessment, the top five zones were as follows: Zone 1 with 10 cases (27.0%), Zone 6 with 21 cases (56.8%), Zone 7 with 12 cases (32.4%), Zone 14 with 12 cases (32.4%) and Zone 15 with seven cases (18.9%).

## DISCUSSION

This prospective multicenter cohort study evaluated outcomes after implantation of the JIIXR Total Knee System in patients requiring TKA for knee arthritis. Results for the 2‐year survivorship demonstrated noninferiority to the 98% performance goal with a 7% margin. PROM results also demonstrated a significant improvement at 2 years compared to baseline. Review of radiographic findings showed that RLLs were observed in over 30% of patients; however, these decreased over time and there were no signs of loosening observed. Collectively, these findings summarize the 2‐year safety and performance of the JIIXR Total Knee System.

Clinical data on earlier‐generation BCR designs report durable long‐term survivorship (82%–89% beyond 22 years), with polyethylene wear as a late failure mechanism [25, 29]. Conversely, newer BCR systems have shown mixed short‐term outcomes with higher early revision rates in three studies (11% and 12%, respectively) [8, 12, 24] alongside a report of promising survivorship of 96.2% for all‐cause reoperation at 2.35 years [34]. Notably, tibial loosening has been a recurring concern in newer designs, underscoring the importance of implant geometry and surgical precision. Against this heterogeneous background, the present data demonstrates good 2‐year survivorship for the JIIXR implant. This result indicates successful preservation and function of the cruciate ligaments, which may reduce the risk of revision due to instability. The low revision rate in our cohort is encouraging; however, cases of early revision due to pain and instability underline the need for further investigation into optimal implant positioning and refinement of the surgical technique.

Our findings for PROMs align with existing literature. Pritchett et al. reported long‐term improvement in American Knee Society scores from 42 to 91 over 23 years; [25] Alnachoukati et al. noted high satisfaction (91% reporting the knee felt normal) without significant differences versus cruciate‐retaining TKAs; [1] and Pelt et al. observed acceptable 3‐year PROMs (mean physical function 45; median pain 3), comparable to traditional TKA [24].

In our cohort, PROMs showed improvement over the 2‐year follow‐up period. Relative to the MCIDs, all KOOS subscales met or exceeded the thresholds [11] by 1 year; EQ‐VAS exceeded its MCID [39] by 3 months; and the FJS‐12 gains between 3 months and 1 year surpassed the reported MCID range [9, 15], with further improvement by 2 years. These results indicate that an anatomical BCR can achieve the level and timing of improvement expected of a high‐performing modern TKA, while future comparative studies are needed to determine any additional benefits over conventional TKA.

With respect to ROM, this cohort had high preoperative flexion (mean 135°), limiting scope for further gains. Extension improved from −4.0° to −1.5°, and total ROM at 2 years did not differ significantly from baseline. This aligns with the well‐established principle that preoperative ROM predominantly determines postoperative ROM—patients with near‐normal baseline flexion typically show minimal additional increases [26]. In accordance with comparative literature, no consistent short‐term ROM advantage has been demonstrated for specific implant constraint patterns [31].

Regarding radiographic findings, 35 cases showed RLLs at 2 years, comparable with previous reports. Christensen et al. [8] reported higher rate of 30% in BCR‐TKAs versus 15% in cruciate‐retaining designs, whereas Biazzo et al. [5] found no significant differences, with rates around 12%–15%. In our cohort, lucencies were small and decreased over time (mean total RLL length per knee 5.1 ± 6.7 mm at 3 months to 2.6 ± 2.3 mm at 2 years), and no radiographic loosening was observed. Early appearance of RLLs is often attributed to micromotion at the cement‐bone interface due to malalignment, residual deformity, or improper bone cuts [37]. Persistent RLLs could pose risks to implant stability [8], emphasizing the need for long‐term follow‐up. In addition, while the mechanical alignment strategy was predominantly used for bone resection in this study, the functional alignment strategy may be more suitable for BCR‐TKA due to its ligament‐preserving nature [17]. Implant alignment could influence stress distribution on the cement‐bone interface and therefore it warrants further investigation. The observed decrease in RLL length is atypical for cemented TKA, where RLLs usually remain stable or progress. Adaptive bone remodelling or gradual cement‐bone interface stabilization might explain the trajectory.

This study has some limitations. First, the single‐arm study design without a parallel control group may introduce selection bias, and relative effectiveness against contemporary TKA designs cannot be inferred. To mitigate this limitation, comparison with a relevant historical reference was included to provide context. Second, the 2‐year follow‐up period is relatively short and extended follow‐up would be beneficial to assess the long‐term performance. Third, procedures were performed by experienced surgeons using mechanical (78/106; 73.6%) or functional alignment (28/106; 26.4%), with severe deformities excluded. The alignment strategy was chosen at surgeon discretion, and the study was neither powered nor prespecified to compare strategies. It's possible this residual confounding could affect the PROMs, ROM, and radiographic findings. Fourth, individual BMI data were not collected (although BMI > 40 kg/m² was excluded), limiting interpretation by body habitus. Finally, this single‐country study used PROMs that can vary by culture or language; EQ‐5D‐5 L value sets differ across countries, and cross‐cultural adaptation studies of FJS‐12 and validation reviews of KOOS [10, 16, 28] indicate that absolute PROM levels and thresholds here may not generalize to other populations.

## CONCLUSIONS

In conclusion, the JIIXR Total Knee System demonstrated a 2‐year survivorship of 97.9%, achieving the prespecified noninferiority criterion relative to a 98% performance goal. Improvements in PROMs and radiographic assessments indicate good early safety and performance. However, longer‐term follow‐up and comparative studies are required to establish the durability and broader clinical impact of these findings.

## AUTHOR CONTRIBUTIONS

All authors contributed to the study conception and design. Ryota Yamagami prepared the draft of the manuscript. Ryota Yamagami, Osamu Nishiike, Ayano Kuwasawa, Katsuhisa Ishii, Daisuke Hamada, Keinosuke Ryu, Shigeshi Mori, and Kotaro Yamagishi collected clinical data. Sarah Megginson performed the data analysis and curated the dataset. Masao Akagi supervised the study, contributed to the study design, interpreted the results, and critically revised the manuscript. All authors reviewed and approved the final version of the manuscript.

## CONFLICT OF INTEREST STATEMENT

Dr. Ryota Yamagami: The University of Tokyo received clinical research fees from Smith & Nephew. The University of Tokyo received donation from Smith & Nephew, Stryker, Kyocera, Zimmer Biomet, Medecta. Dr. Yamagami reports payment or honoraria for lectures, presentations, speakers bureaus, manuscript writing or educational events from Smith & Nephew, Stryker, Teijin Nakashima Medical, Kyocera. Dr. Osamu Nishiike: The Kushiro Sanjikai Hospital received clinical research fees from Smith & Nephew. Dr. Nishike reports payment or honoraria for lectures, presentations, speakers bureaus, manuscript writing or educational events from Smith & Nephew, Depuy Synthes, MicroPort Orthopedics, United Orthopedics; support for attending meetings and/or travel from Smith & Nephew. Dr. Ayano Kuwasawa: The Saitama Cooperative Hospital received clinical research fees from Smith & Nephew. Dr. Kuwasawa reports payment or honoraria for lectures, presentations, speakers bureaus, manuscript writing or educational events from Smith & Nephew, Zimmer Biomet; support for attending meetings and/or travel from Smith & Nephew, Zimmer Biomet. Dr. Katsuhisa Ishii: The Toho University received clinical research fees from Smith & Nephew. The Toho University received donation from Smith & Nephew, Zimmer Biomet. Dr. Daisuke Hamada: The Tokushima University received clinical research fees from Smith & Nephew. The Tokushima University received donation from Smith & Nephew. Dr. Hamada reports payment or honoraria for lectures, presentations, speakers bureaus, manuscript writing or educational events from Smith & Nephew, Kyocera. Dr. Keinosuke Ryu: The Nihon University and received clinical research fees from Smith & Nephew. The Nihon University and The Fukushima Medical University received donations from Smith & Nephew. Dr. Ryu reports payment or honoraria for lectures, presentations, speakers bureaus, manuscript writing or educational events from Smith & Nephew; support for attending meetings and/or travel from Smith & Nephew. Dr. Shigeshi Mori: The Kindai University received clinical research fees from Smith & Nephew. The Kindai University received donations from Smith & Nephew, Kyocera, Zimmer Biomet. Dr. Mori reports consulting fees from Smith & Nephew; payment or honoraria for lectures, presentations, speakers bureaus, manuscript writing or educational events from Smith & Nephew; support for attending meetings and/or travel from Smith & Nephew. Dr. Kotaro Yamagishi: The Kindai University received clinical research fees from Smith & Nephew. The Kindai University received donations from Smith & Nephew, Kyocera, Zimmer Biomet. Dr. Yamagishi reports consulting fees from Smith & Nephew; payment or honoraria for lectures, presentations, speakers bureaus, manuscript writing or educational events from Smith & Nephew. Sarah Megginson: An employee of Smith & Nephew and receive a salary from the company. Dr. Masao Akagi: The Kindai University received clinical research fees from Smith & Nephew. The Kindai University received donations from Smith & Nephew, Kyocera, Zimmer Biomet. Dr. Akagi reports consulting fees from Smith & Nephew, Kyocera; payment or honoraria for lectures, presentations, speakers bureaus, manuscript writing or educational events from Smith & Nephew, Kyocera.

## ETHICS STATEMENT

This study was conducted in accordance with the Declaration of Helsinki and was approved by the Institutional Review Board of The University of Tokyo (approval No. 2018098NI), Kushiro Sanjikai Hospital (approval No, N/A), Saitama Cooperative Hospital (19‐02‐01), Toho University Ohashi Medical Center (H19001), Tokushima University Hospital (3484), Nihon University Itabashi Hospital (RK‐190614‐01), Kindai University Nara Hospital (539), Kindai University Hospital (31‐010). Written informed consent was obtained from all participants.

## Data Availability

The authors have nothing to report.
